# “One Teabag Is Better than Four”: Participants Response to the Discontinuation of 2% PRO2000/5 Microbicide Gel in KwaZulu-Natal, South Africa

**DOI:** 10.1371/journal.pone.0014577

**Published:** 2011-01-21

**Authors:** Mitzy Gafos, Misiwe Mzimela, Hlengiwe Ndlovu, Nkosinathi Mhlongo, Yael Hoogland, Richard Mutemwa

**Affiliations:** 1 Africa Centre for Health and Population Studies, University of KwaZulu-Natal, KwaZulu-Natal, South Africa; 2 City University, London, United Kingdom; 3 London School of Hygiene and Tropical Medicine, London, United Kingdom; 4 The Microbicides Development Programme Team, Microbicides Development Programme, Clinical Trials Unit, Medical Research Council, London, United Kingdom; Dalhousie University, Canada

## Abstract

**Introduction:**

The Microbicides Development Programme evaluated the safety and effectiveness of 0.5% and 2% PRO2000/5 microbicide gels in reducing the risk of vaginally acquired HIV. In February 2008 the Independent Data Monitoring Committee recommended that evaluation of 2% PRO2000/5 gel be discontinued due to futility. The Africa Centre site systematically collected participant responses to this discontinuation.

**Methods:**

Clinic and field staff completed field reports using ethnographic participant observation techniques. In-depth-interviews and focus group discussions were conducted with participants discontinued from 2% gel. A total of 72 field reports, 12 in-depth-interviews and 3 focus groups with 250 women were completed for this analysis. Retention of discontinued participants was also analysed. Qualitative data was analysed using NVivo 2 and quantitative data using STATA 10.0.

**Results:**

Participants responded initially with fear that discontinuation was due to harm, followed by acceptance after effective messaging, and finally with disappointment. Participants reported that their initial fear was exacerbated by being contacted and advised to visit the clinic for information about the closure. Operational changes were subsequently made to the contact procedures. By incorporating feedback from participants, messages were continuously revised to ensure that information was comprehensible and misconceptions were addressed quickly thereby enabling participants to accept the discontinuation. Participants were disappointed that 2% PRO2000/5 was being excluded as a HIV prevention option, but also that they would no longer have access to gel that improved their sexual relationships with their partners and assisted condom negotiations. In total 238 women were discontinued from gel and 185 (78%) went on to complete their scheduled follow-up period.

**Discussion:**

The use of qualitative social science techniques allowed the site team to amend operational procedures and messaging throughout the discontinuation period. This proved instrumental in ensuring that the discontinuation was successfully completed in a manner that was both understandable and acceptable to participants.

**Trial registration:**

Current Controlled Trials. ISRCTN64716212.

## Introduction

### Clinical trials

Clinical trial sponsors, investigators and participants generally plan for a clinical trial to continue until scheduled completion. However, trials may be prematurely discontinued for a number of reasons. As part of the clinical trial governance and monitoring process, trial data is systematically reviewed for safety and progress towards primary endpoints by an Independent Data Monitoring Committee (IDMC). The IDMC has the mandate to recommend discontinuation of the clinical trial, or part thereof, as it deems fit on the following basis: 1) if the new treatment demonstrates significant effectiveness, 2) if the new treatment demonstrates safety concerns or harm to the participants; or 3) if it is clear that efficacy will not be demonstrated (futility) [1]–[3].

### Microbicide clinical trials

Microbicides are experimental products which may reduce the risk of HIV infection for women during sexual intercourse. To date seven microbicides have entered clinical trials evaluating product effectiveness [4] and three trials evaluating two products have been prematurely halted. Family Health International trials of SAVVY microbicide were stopped in Ghana in 2005 due to declining HIV incidence and in Nigeria in 2006 on the basis of product futility [5], [6]. The following year a CONRAD trial evaluating Cellulose Sulphate microbicide was discontinued on the basis of potential harm [7]. FHI subsequently halted its trial of Cellulose Sulphate as a precautionary measure without any indication of harm [8]. The initial closure of the CONRAD Cellulose Sulphate trial on the basis of a higher number of HIV infections in women using the product received extensive media coverage in KwaZulu-Natal [9] although the final trial results showing that the product did not cause harm but was futile, received minimal media coverage [10].

### Microbicides Development Programme

The Microbicides Development Programme (MDP) was established to develop safe, effective, acceptable and affordable microbicides (http://www.mdp.mrc.ac.uk/). MDP is a partnership of 16 African and European research institutions. The UK Medical Research Council (MRC) sponsored the MDP301 international, multi-centre, randomised, double-blind, placebo-controlled, phase III clinical trial that aimed to evaluate the safety and effectiveness of 0.5% PRO2000/5 and 2% PRO2000/5 microbicide gels in reducing the risk of vaginally-acquired HIV infection. The design of the overall trial and the integrated social science component, as well as the trial results have been published elsewhere [11]–[13]. The MDP301 trial was conducted at six clinical trial centres in South Africa, Tanzania, Uganda and Zambia. The first site commenced enrolment in October 2005. At enrolment women were randomly assigned to receive 0.5% PRO2000/5, 2% PRO2000/5 or a placebo gel. The trial was double-blinded, so neither trial staff nor participants knew to which gel group participants were allocated. Women enrolled in the trial were instructed to insert 2 ml of gel vaginally up to one hour prior to sex from a pre-filled applicator. Participants received monthly HIV prevention counselling which included support in negotiating condom use. They were required to return to the clinics every four weeks for a total of 52 weeks (104 weeks at the Uganda site). At the end of their follow-up period all unused gel was collected.

On the 8^th^ February 2008, the IDMC for the MDP301 protocol met for the sixth time and reviewed data collected on 7,735 women available by 15^th^ January across all six sites. The IDMC recommended that the evaluation of 0.5% PRO2000/5 in comparison to the placebo gel continue. However, they recommended that evaluation of 2% PRO2000/5 gel should be discontinued as there was ‘no more than a small chance of it showing protection against HIV infection’ [14]. Following the IDMC recommendation, the MDP Trial Steering Committee (TSC) met on 11^th^ February 2008 to review the IDMC recommendations. After careful consideration of the operational, scientific and ethical issues involved, the IDMC recommendations to stop evaluation of 2% gel and continue evaluating 0.5% gel were accepted. Participants were to be discontinued from 2% gel use as soon as practically possible, they would not be randomised back into the trial in order to maintain validity of the randomisation schedule and there were no clinical or ethical benefits of providing placebo gel to women without it being used as a comparator for an investigational product.

At the time of the discontinuation, the investigators remained blinded and were not provided with the data on which the IDMC made their decision. However, the final trial results demonstrated that the conditional power for significant benefit from the 0.5% PRO 2000 gel was sufficient to warrant trial continuation [13].

### Africa Centre for Health and Population Studies MDP site

The Africa Centre for Health and Population Studies was one of six MDP301 clinical trial sites (www.africacentre.ac.za). The Centre is located in a predominantly rural part of the Umkhanyakude District of KwaZulu-Natal, South Africa [15]. The Africa Centre joined the MDP network in 2003 but had a well established institutional community advisory board (CAB) and long-standing relationships with a broad range of stakeholders from its inception in 1998. The standard of care package offered to MDP301 trial participants and their partners was extensively negotiated with the CAB and other relevant stakeholders, as well as being revised with trial participants after enrolment commenced [16], [17]. As part of these negotiations, it had been agreed that before the end of follow-up, trial participants would be informed about the range of vaginal lubricants available locally, but that alternative lubricants would not be distributed by the research team. This decision was based on a number of factors; 1) there would not be long term clinical follow up after the trial in case of adverse reactions to lubricants, 2) there was emerging evidence that over-use of one of the locally available lubricants could disrupt the vaginal mucosa, and 3) that it could be extremely difficult to prevent assumptions in the community that over-the-counter vaginal lubricants had anti-HIV properties. However, at the end of follow-up each participant had a close-out session which included a full explanation of trial timelines, result scenarios, plans for unblinding participants and disseminating results, as well as plans for post-trial access in the eventuality that the product had been found safe and effective.

The Microbicide Study Coordinating Committee (MSCC) served as the management committee for the MDP301 protocol at the Africa Centre. The MSCC included the trial investigators as well as coordinators responsible for the clinical, counselling, social science, data, recruitment, retention, community liaison, laboratory and pharmacy components of the trial. The MSCC met on 12^th^ February to discuss the IDMC recommendation and established procedural guidelines for the discontinuation of trial participants from the 2% gel arm. Discontinuation was planned to commence on the 14^th^ February 2008 across all sites in line with the public release of the discontinuation. The top priorities of the discontinuation plan at the Africa Centre were: 1) to disseminate the information quickly and comprehensively to trial participants, community members and stakeholders; and 2) to discontinue women from 2% PRO2000/5 gel.

The MSCC developed a script to explain the key messages (available on request). The script was reviewed during a series of workshops conducted with staff, participants and community members. Participants were mainly drawn from the participant advisory group which was a fluid group of former and current participants who were invited to attend meetings on various topics through an established participant at each of the three clinics. Community members were mainly drawn from the institutional CAB which included community elected representatives who were briefed on all aspects of research conducted at the Africa Centre but were not MDP trial participants. The workshops provided a platform to ensure that the messages utilized appropriate language and were comprehensible. The script was presented at meetings and community events, as well as via local press and radio shows. Audio recordings were made of the script and played in the clinics. Underpinning the information dissemination process was a monitoring strategy designed to collect participant and community responses to the discontinuation of the 2% gel. The purpose of the monitoring exercise was to ensure that information dissemination procedures were working effectively, disseminated information was accurately comprehended, and any adverse responses to the discontinuation were detected and addressed immediately. As a result of the monitoring strategy, the script and audio recordings were regularly reviewed and revised to incorporate additional questions from participants and community members and to address local misconceptions that were reported to the MSCC, who met daily during this period.

The MSCC also established a daily reporting mechanism for the clinic teams to report the number of trial participants who had been given the information and the number of women discontinued from 2% gel. MRC supplied a list of trial numbers that had been allocated to the 2% gel arm of the trial. By the 14^th^ February 2008, 1,056 women had been enrolled in the trial at the Africa Centre, with 353 randomised to the 2% gel arm. Of those on 2% gel, 238 were still within the 52 week follow up period at the time of discontinuation. All participants on 2% gel were contacted by telephone where possible or visited at home. At the time of gel discontinuation, participants underwent pregnancy testing, HIV counselling and testing and a genital examination which included the collection of samples to test for sexually transmitted infections (STIs). Women who were discontinued from gel use were invited to remain in the study attending only the quarterly clinical visits instead of also attending the monthly gel accountability visits. However, women discontinued from 2% gel were not tracked if they did not return for a quarterly visit after they had been discontinued as they no longer required clinical monitoring. This paper presents the responses of participants at the Africa Centre to the discontinuation of 2% PRO2000/5 gel.

## Methods

The clinical trial protocol included approval for ethnographic participant observations within the trial. When the 2% gel was discontinued the MSCC members developed monitoring systems to evaluate responses to the discontinuation using these ethnographic techniques. This included the development of a field report that all MDP clinic and field staff members were required to complete on a daily basis. All clinic and field staff regularly presented information about HIV and the MDP study in the community and therefore had undergone training in the completion of field reports to capture community questions and feedback. The reports systematically documented anonymous information that trial staff were either told or overheard at the clinics or in the community. These field reports summarised the participants' responses to the discontinuation, their level of comprehension of disseminated information and questions posed by them. A total of 72 field reports were submitted over 16 days from the 14^th^ to 29^th^ February 2008 documenting views of 216 trial participants regardless which arm of the study they were randomised to. The field reporting stopped at the end of the month as the majority of women on 2% gel had been discontinued and data saturation had been reached. The field reports were transcribed in English.

The trial protocols social science component included in-depth interviews (IDI) and focus group discussions (FGDs) with trial participants. Participants in the IDIs and FGDs provided written informed consent. Twelve IDIs and 3 FGDs were conducted with women discontinued from the 2% gel arm. Women in the IDIs were aged between 19 and 64 years and were discontinued from gel use between 14^th^ February and 27^th^ March. The FGDs involved 22 discontinued women aged between 19 and 57 years. The IDIs and FGDs were conducted in isiZulu and were recorded, they were transcribed in isiZulu and translated into English.

All of the transcripts of field reports, IDIs and FGDs, were imported into the qualitative data analysis software NVivo 2 (QSR International Pty Ltd. Version 2, 2002) and coded according to a matrix of themes that had been predefined for all community liaison data collection. The coding themes were developed on the basis of findings from formative research conducted during earlier feasibility and pilot studies. The broad coding was based on who had provided the data (women screened, women enrolled, male partners, women or men from the community, members of formal community groups, or community advisory board members) and the topic of discussion (about the research institution, personal benefit or loss due to participation, MDP study policies, clinical procedures, or gel). The clinic attendance of women discontinued from the 2% PRO2000/5 gel at quarterly visits was also monitored and reasons for withdrawals were documented. This information was recorded on trial case record forms which were double entered using an Access application with a SQL Server. The quantitative data was analysed using STATA 10.0 (Stata Corporation, College Station, Texas, USA). At the Africa Centre the MDP301 clinical trial was approved by the South African Medicines Control Council (N2/19/8/2) and the University of KwaZulu-Natal Biomedical Research Ethics Committee (T111/05). These bodies also reviewed the revised protocol and information sheets at the time of the discontinuation. Pseudonyms are used in this paper to maintain participants' anonymity.

## Results

The qualitative analysis highlighted four key topics; 1) participants responses to the procedures used during discontinuation, 2) participants interpretation of the discontinuation messages; 3) participants responses to the discontinuation; and 4) participants responses regarding informing their partners. We also present quantitative data on how many women decided to remain in follow-up to the end of the scheduled period after being discontinued from 2% PRO2000/5 gel.

### Participant responses to discontinuation procedures

Initially participants randomised to the 2% gel arm were contacted by phone if a current phone number was available or during home visits if a phone number was unobtainable. They were informed that there was new information about the trial and asked to visit the clinic as soon as possible instead of waiting for their next scheduled visit. This was driven by a number of factors; firstly the nurses and counsellors were considered the most appropriate staff members to explain the information comprehensively and could take time to address all questions; secondly there was a concern that by providing only partial information at the initial contact, participants may not visit the clinics for more information or may not return with the unused gel applicators; and thirdly the tracking team consisted of only 4 staff members and therefore the workload of contacting all participants was already arduous. However, within the first few days, feedback from participants indicated that the resulting suspense created significant apprehension among them. Participants immediately assumed that the gel must have caused harm. For instance:

*‘I thought that it (gel) had some danger because he (study team member) called twice. I said is there anything that has happened and he said yes and I asked is it so bad and he said I shouldn't panic, but I was so scared.’* Samke, 46 year old, IDI.

The MSCC changed the procedure on the third day of dissemination. Fieldworkers contacting participants were then provided with an abridged script to explain basic information about the discontinuation, before advising the participant to visit the clinic for further details. In order to allay fears of harm, the key discontinuation message was headlined in the script. The majority of participants responded positively to this procedure as they said that learning about the discontinuation directly from the study team was preferable to hearing the news in the community or media.

Less than half of the telephone numbers provided by participants were operational and therefore the majority were visited at home. Some participants reported that even though they had previously agreed to be visited at home they were apprehensive when they were visited by the staff vehicle, especially if they had not disclosed trial participation to family members or neighbours. The HIV and non-HIV related research work of the Africa Centre had been continuously explained in community settings over the previous 10 years. Despite this, and a continuous presence of Africa Centre vehicles, remnants of a rumour remained that associated visits by Africa Centre vehicles with a HIV-positive person being in the household. In an attempt to mitigate this stigma, study participants were encouraged to consider these issues before enrolment and to prescribe in considerable detail the best modes of contact and ‘reasons’ for contact when a staff member met a family member, work colleague or neighbour. Some participants requested that research staff park at a discrete distance and walk instead of drive up to their houses if home visits were necessary. However, the majority of women did not define specifications, and for a minority this appeared to be because they did not expect to require a home visit. Although some participants would have preferred to have been contacted by phone, participants were only contacted at home if the phone number provided by participants was no longer in use. A solution to the dilemma of wanting to hear the information from the study team as soon as possible but not wanting to be contacted at home when an active phone number was not available was never resolved. However, the MSCC did subsequently increase procedures that monitored if clinic staff checked contact details with participants at every monthly visit. The team also suggested that in subsequent trials it could be helpful to insist on an initial visit away from the clinic prior to enrolment to ensure that the trial participant and staff member developed and tested acceptable modes of contact.

### Participants' interpretation of the discontinuation messages

The site team recognised that there were specific challenges to explaining why the higher concentration gel (2%) was being discontinued and the lower concentration gel (0.5%) was continuing to be evaluated. The field reports documented how participants explained this among themselves. One participant who was a traditional healer explained that in traditional medicine the aim is to use the weakest concentration possible. She explained to other participants that she was not at all surprised at the discontinuation of the higher concentration PRO2000/5 and the continued evaluation of the lower concentration. One group of participants likened the discontinuation of 2% gel and continuation of 0.5% gel to having two cups of hot water, both with one teaspoon of sugar: but, one cup with one teabag and the other cup with four teabags. The group explained the better cup of tea would be the one brewed with a single teabag (0.5% gel), not the one with four teabags (2% gel).

Despite these examples, many participants said that they had placed more hope on the higher concentration PRO2000/5 gel and some participants said they had lost hope in the 0.5% gel as a result of the 2% discontinuation. Field reports observed that participants indicated that:

*‘…they would now pray four times harder for 0.5% gel to work (against HIV) as it is four times weaker than the 2% gel…’* Nozipho, participant in 40's, field report.

*‘They have doubts for the 0.5% PRO2000/5 gel… They said “we are holding our breaths” as there is now less hope that the 0.5% will work.’* Smisiwe, participant in 40's, field report.

The field reports also identified three misconceptions which only emerged in a handful of situations but could have spread if not attended to; 1) the first was that if the IDMC had been able to see that 2% PRO2000/5 was not likely to prevent HIV infection, by recommending that 0.5% gel continue the IDMC must have seen that 0.5% gel was effective against HIV; 2) the second was that because the 2% gel was stopped due to futility and not because of harm, there was evidence that 2% PRO2000/5 was safe and this was also used as an indicator that 0.5% PRO2000/5 must be safe; 3) the third was that the whole trial had been un-blinded as participants questioned how the site staff, who they knew were blind to gel allocation, could identify women on 2% gel. Whilst these interpretations demonstrate a complex understanding of the scientific issues, they lead to incorrect assumptions that the 0.5% gel is effective, safe and unblinded. Messages were quickly revised or developed to update the script to avoid the spread of these misconceptions. As a result, these reports dissipated within the first week of the discontinuation process.

### Participant responses to the discontinuation

Overall most participants were calmly accepting of the discontinuation and the reasons for it. Most of the participants acknowledged that they were told at the beginning of the trial that the microbicide gels were being tested and it was not known if they were effective against HIV, as the following quote demonstrates:

*‘It was explained to me that it (gel) was still being tested and if it doesn't work it will end like this and you will stop. You cannot continue with something that doesn't work…’* Bonisiwe, 33 year old, FGD.

However, despite understanding that PRO2000/5 was an investigational product, many participants had hoped that the gel would prove effective against HIV and hence protect them. Thus, when the discontinuation was announced most women were deeply disappointed as expressed by the following participants:

*‘We had hope because this gel was something of a high note and we hoped that it (gel) might work and thereafter we could get it from the chemist. I had that hope.’* Cebile, 46 year old, IDI.

*‘…they (study clinic staff) explained to us…that it was not known whether this thing (gel) will protect us against diseases (HIV). But because it was tested on me as a person I put my trust in this thing…. but now if it is found that it didn't help me, hawu (exclamation of sadness)!’* Thembi, 33 year old, FGD.

Some participants said that their initial response to the news that the 2% gel arm had been discontinued was fear, with the immediate conclusion that the gel had been found to be harmful. Many linked this response to the initial reports of the Cellulose Sulphate microbicide trial being closed due to safety concern, which had been heavily publicised in the community. One participant said in an IDI:

*‘We entered (the clinic room) with another lady and they (clinic staff) explained…we looked at each other and thought that the gel might be really harmful. I was just thinking for myself. Everyone was thinking that but we didn't ask each other…I told myself that it (gel) is harmful, in other words I'm infected. Then the blood was taken and there is nothing that is left with me (I'm HIV negative).’* Lindiwe, 52 year old, IDI.

With consistent explanations from the study team, participants' fears were gradually allayed. However, some participants expressed concern that community members may assume that the gel had been discontinued due to increased risk of HIV infection, as had been the initial report of the Cellulose Sulphate trial. Participants reported that they promoted their participation in the trial as a sign of their negative HIV status to friends and neighbours. They feared that by stopping gel use they may be assumed to have sero-converted as explained in the following quote:

*‘I think the community will look at us badly because even if you explain that we have stopped this gel because the gel doesn't work, not because it is harmful…the community will tell itself that the gel has been stopped because we are infected.’* Sizakele, 23 year old, IDI.

Nonetheless, overall there was a general sense of relief among participants that the discontinuation was due to futility rather than harm.

### Relating information to male partners

Only a few male partners of trial participants visited the clinics for information about the discontinuation. This meant that the burden of responsibility to explain the discontinuation rested with the participants. Most women on 2% gel reported that they did not tell their partners about the discontinuation until after they had completed the discontinuation visit. In this way they said they felt more confident that they could explain the discontinuation. They also used the HIV test result and the pelvic examination as ‘proof’ that the 2% gel was being discontinued for futility rather than harm. Participants had clearly discussed their results of the HIV tests and pelvic examinations between them and used their collective experience as additional evidence. One participant explained:

*‘I explained to him (partner) that the gel was tested and found not to prevent HIV as it was expected and he said it is okay if it (gel) hasn't done any harm to us. I said that there is nothing (no harm) because we (participants) were examined and nothing was found.’* Tholakele, 49 year old, FGD.

As has been the experience with most investigational microbicide gels [18]–[26], most of the participants in the IDIs and FGDs said the gel had increased their sexual pleasure and improved their relationships. Some said that their partners were now less likely to be unfaithful as a result of them using the gel. Therefore, when discontinuation of the 2% gel arm was announced the concern about the impact that this would have on relationships and sexual experiences was dominant. This concern was evident in these reports:

*‘The lady (study field staff) said I was invited to the clinic. I asked what have I done and she said nothing…take all your gels and bring them to the clinic. I thought they (clinic staff) were going to add some gels because they gave me a lot and I (often) returned all of them empty. The lady said I will not return home with the gels and I said can we please hide some, but she said no.’* Beauty, 51 year old, FGD.

*‘The participant said gel helped her in her relationship since her partner used to cheat on her (before). She was worried about what she is going to use now if she has to return all her gel back. She returned home to collect all her gels and brought them back to the clinic. She was so angry you could see that on her face.’* Khetiwe, 52 year old, field report.

Many participants reported an increase in condom use since joining the study and using gel. Many said the positive impact the gel had on their sexual pleasure made condom use more tolerable to both them and their partners. The discontinuation raised concerns that they would no longer be able to negotiate condom use with their partners:

*‘I collected condoms from here in the (study) clinic. I explained to him that I use this gel not because it would protect us against disease…they (investigators) do not have proof that the gel protects. (He) agreed and said that he would use condoms. Before that he did not use condoms.’* Lungeleni, 38 year old, IDI.

*‘Gel made my partner behave well and he respected me by wearing condoms.’* Lindiwe, 52 year old, IDI.

There had always been a lingering apprehension among participants that once they completed their scheduled follow up period in the trial that their relationships and their ability to negotiate condom use would be impacted. During the 2% discontinuation women most recently enrolled voiced this concern most loudly. This could have been because women at a later stage in their follow up period had already started to come to terms with having to stop using gel at the end of the study.

The majority of participants on 2% gel had disclosed gel use to the male partners (57% of 325 women who attended their week 4 visit). Participants felt that dealing with the discontinuation was more complicated for trial participants who had not disclosed gel use to their partners. They felt that the partners were likely to notice a reduction in sexual pleasure when the women stopped using the gel and that the women would find it difficult to explain the change. A study fieldworker entered the following in her monitoring report after a conversation with a participant:

*‘Her partner had asked her what she is using that makes her “nice” in bed and because she had not disclosed gel use she answered that she does not know. Now (that she will no longer be using gel) she was concerned that her partner will feel the difference and accuse her of cheating and she might even need to disclose to him that she had been using gel and she doesn't know how he is going to take that explanation.’* Nonhlanhla, 20 year old, field report.

Because of all these concerns some participants discontinued from 2% gel asked to be randomised into the remaining two arms of the trial, so that they could continue accessing the gel. Other participants asked to be given the placebo gel. The study team were under constant pressure to explain why both cases were not possible, for scientific and ethical reasons.

Due to these concerns participants in the FGDs believed that most women discontinued from the 2% gel would not return their unused gel. However, due to consistent and effective messaging, over 99% of unused 2% gel applicators were returned by participants who were discontinued from gel use.

### Attendance after discontinuation

For the purpose of these analyses, ongoing attendance at the study clinics after gel discontinuation was used as a proxy measure of acceptance that the information provided was accurate and that the gel had not caused harm. This is based on the hypotheses that if women had lost trust or faith in the study they were likely to decide to no longer attend the study clinics.

Of the 238 participants randomised to the 2% gel arm and still within the follow up period by February 2008, 209 (88%) were located and discontinued within 3 weeks of February 14^th^. An additional 15 participants were discontinued over a period of 4 to 20 weeks after 14^th^ February. The remaining 14 women had defaulted on their follow up visits prior to the discontinuation and were never located.

Before February 2008, 101 (88%) of the 115 women in the 2% PRO2000/5 gel arm had completed the scheduled trial follow-up, 7 had defaulted and 7 had withdrawn from the trial. Of the 238 women discontinued from the 2% PRO2000/5 gel arm, 185 (78%) completed the scheduled follow-up period, 14 had defaulted prior to the discontinuation and were never located (as mentioned above), 2 women died of unrelated conditions before completion, 34 defaulted after the discontinuation visit, and 3 withdrew from the trial. In fact, 20 participants had only had one follow-up visit by the time of discontinuation, yet 12 of these completed their remaining eleven months of follow-up. This high level of follow-up was attained without any tracking procedures for women discontinued from 2% gel.

Although the field reports did not exclude women randomised to 0.5% or placebo gel, this paper focuses on accounts from women who were discontinued from 2% gel. All enrolled women (regardless of gel group) were asked to provide written consent to continue in the trial after the discontinuation had been explained. Overall women in the other trial arms were relieved that evaluation of 0.5% gel was continuing, no-one withdraw from the 0.5% and placebo groups as a result of the discontinuation, and trial retention and gel adherence was not affected in anyway.

## Discussion

This paper set out to systematically review responses of participants to the discontinuation of the 2% PRO2000/5 gel arm of the MDP301 clinical trial at the Africa Centre in February 2008. A range of qualitative methods were used which included field reports, IDIs and FGDs, in order to monitor consistency between solicited and unsolicited information. The qualitative data indicated that the participants were generally accepting of the discontinuation. Quantitative retention data was then used, as a proxy measure, to monitor consistency between verbal accounts of acceptance and ‘active’ demonstration of acceptance in terms of remaining engaged with the study. The fact that 78% of women discontinued from gel use completed their scheduled follow-up visits supports the level of acceptance observed in the qualitative data.

The MDP301 protocol included a substantial social science component and the application of these qualitative techniques during the discontinuation period was critical in informing operational procedures. Feedback from participants within the first 2 days of the discontinuation resulted in the MSCC changing the initial contact procedures by the tracking team. This feedback also facilitated the iterative review of messages and allowed the team to address misconceptions at their formative stage before they spread and became entrenched. Initial reports also identified that participants were concerned about the potential reaction that their communities may have to the news given the previous media coverage of the Cellulose Sulphate trial being initially stopped due to a risk of increased harm. This highlighted the need to hasten and intensify community level messaging and to prioritise areas that were known to have significant concentrations of participants. The utilisation of feedback mechanisms to inform operational procedure had always been a core component of the trial protocol and proved invaluable during this unexpected event.

The qualitative data indicated that participants comprehended the information explaining why the 2% gel arm had been discontinued. The educational components of the trial and the regularity with which the key trial messages had been repeated throughout the trial evidently enabled participants to understand the discontinuation. For example participants framed the news within the context of understanding the investigational nature of the gels and the double-blind trial design. In addition, the comprehensibility of the discontinuation messages was enhanced by engaging a broad spectrum of staff as well as participants and community members in the development of the messages. The messages were continuously improved through the daily iterative review process. This ensured that the messages drew on real life scenarios that had local meaning in isiZulu. For example the explanation that “one teabag is better than four” was incorporated to explain that a stronger concentration is not necessarily better than a weaker concentration at achieving a desired outcome. This assisted in explaining why 2% PRO2000/5 was being excluded as a HIV prevention option, but there was still a chance that 0.5% PRO2000/5 may prove effective as a HIV prevention option. The ability of the trial team to develop messages in this manner was supported by the fact that the sponsors provided key messages for site staff but devolved the responsibility of developing local messages for participants and community members to the site investigators. This devolution of responsibility had been established across the network from the beginning of the trial as the sponsors acknowledged the differences between the clinical trial sites in the network and the different needs of their local populations.

The utilisation of the qualitative methods also allowed the study team to quickly gauge the extent to which messages were misinterpreted or extrapolated to other parts of the trial. For example, participants interpreted the fact that 2% gel was being discontinued due to futility to mean that it was safe. They also extrapolated this to mean that 0.5% PRO2000/5 must also be safe. These reports highlighted that the messages did not include the scientific evidence based principle in which there was no evidence 2% was harmful yet there was not evidence it was safe. It was crucial for the team to explain this evidence based principle not only to women discontinued from the 2% gel but to all participants so as they did not think that the 0.5% gel was ‘safe’ thereby undermining the key message that the trial was evaluating both effectiveness and safety.

Many participants reported that their initial response to the news was one of fear and apprehension at the risk of harm. The fact that participants used their own health status as confirmation that the gel was discontinued due to futility not harm did not prove problematic as there were no sero-conversions or genital abnormalities diagnosed at the discontinuation visits. However, if there had have been, this link would have been very problematic. This association stemmed from two issues; firstly the closure of the Cellulose Sulphate trial the previous year, and; secondly a lack of knowledge regarding all the circumstances under which a trial, or part thereof, may be prematurely discontinued. The participant information sheet only explained that participation in the study could be stopped prematurely ‘by authorities responsible for running the trial’. The role of the IDMC was not comprehensively explained to all MDP participants until the Cellulose Sulphate study was closed. However, the fact that a trial, or part thereof, could also be discontinued due to effectiveness or futility was never fully explained prior to the 2% discontinuation. This lack of prior knowledge unduly caused anxiety for participants at the time of the discontinuation. Whilst it is important for participants to understand all aspects of the trial before consenting to participate, there is always a risk of information overload which can result in the key messages being confused or forgotten. The trial team consequently decided to introduce comprehensive explanations about the role of the IDMC and the circumstances under which a trial may be discontinued to subsequent enrolees as modular components in the ongoing information sessions that are delivered throughout the course of the trial.

Overall participants were accepting of the news, but were disappointed. Whilst participants clearly understood the investigational nature of the trial gels, their dire need for additional HIV prevention options meant that they ‘hoped’ that the gel would prove to be a HIV prevention option that they could use in the future. However, what was also obvious from the reports was that the disappointment went beyond merely being disappointed that 2% PRO2000/5 was being excluded as a potential HIV prevention option. For most participants, gel use had resulted in improvements in their relationships and sexual experiences. Gel use had also assisted some women in negotiating the use of condoms with their partners. Consequently, losing the ability to use gel had much broader impacts on participants' lives. Basically, participants perceived discontinuation as a threat to the present favourable status of their relationships, regardless of the scientific justification that investigators provided for the decision to discontinue.

As previously stated, the retention data served as a proxy measure for ‘active’ demonstration of acceptance of the discontinuation. However, as the qualitative data was collected within the first few months of discontinuation, it was not possible to ascertain from this analysis why so many women continued to attend their three-monthly follow-up visits after their discontinuation from gel. One option is that they wanted to continue to access the high standard of care offered by the research clinics. Whilst HIV counselling and testing is widely available in the community, pelvic examinations and STI tests are not routinely conducted. A second option may be that given their reports of using their HIV tests and pelvic examinations as a way to ‘confirm’ that the gel had not ‘harmed’ them, they used the quarterly visits to continue to check their health status. The site was only able to facilitate this continued follow-up of participants because only one arm of the trial was discontinued but the rest of the trial continued to its scheduled completion 18 months later. If participants did use the continued follow-up visits as confirmation that the gel had not caused harm, it raises the question of how participants respond to trials that close in their entirety prematurely in terms of their own perception of risk.

### Conclusion

The implementation of the monitoring systems proved instrumental in ensuring that the discontinuation was successfully completed in a manner that was both understandable and acceptable to participants. This paper draws on the experiences of only one of six research centres involved in this multicentre trial and therefore the findings do not represent the diverse populations of other settings. However, a number of concrete lessons for future trials emerge from these analyses such as the benefits of ongoing feedback mechanisms, devolved responsibility for local messaging, gauging the accuracy with which messages are understood and incorporating extended information about the role of IDMC's. These findings also support the recommendation in the Good Participatory Practice guidelines that prior to trial implementation, research teams need to develop plans for early termination of trials in consultation with relevant stakeholders [27]. Researchers should continue to document the process of early trial termination and participant's experiences of it, so as these examples can be used in future trials to better prepare participants and community structures for the potential of early trial termination [7].

More generally, these analyses highlight that enrolment in a clinical trial is a dynamic experience for participants; they actively engage in the trial, constantly learn new information, and continuously re-evaluate the benefits of participation within the context of their broader lives. This experience is also influenced by changes in trial circumstances, such as premature discontinuation of a study arm. Consequently changes to clinical trials have to take account of this dynamic experience to ensure that the circumstances are still acceptable to participants and the community. The Africa Centre sites experience of the discontinuation of the 2% PRO2000/5 gel highlights the multiple benefits of incorporating qualitative social science techniques in the trial protocol, not merely to assess sexual behaviour, but to inform operational procedures, messaging and to monitor and evaluate the dynamic experience of participants.
